# A prognostic nomogram for colorectal cancer based on ubiquitin-specific protease 21 expression: a retrospective cohort study

**DOI:** 10.3389/fmolb.2026.1764916

**Published:** 2026-03-16

**Authors:** Hua Chen, Tianhao Lao, Tong Shen, Yongqiang Wang, Jie Yang

**Affiliations:** Department of General Surgery, Affiliated Kunshan Hospital of Jiangsu University, Kunshan, China

**Keywords:** colorectal cancer, nomogram, overall survival, prognosis, USP21

## Abstract

**Background:**

Colorectal cancer (CRC) remains a leading cause of cancer-related mortality worldwide, and substantial prognostic heterogeneity exists among patients with similar TNM stages. Ubiquitin-specific protease 21 (USP21) has been implicated in tumor progression across multiple malignancies; however, its prognostic value in CRC has not been fully elucidated. This study aimed to evaluate USP21 protein expression in CRC tissues and develop a USP21-based nomogram for individualized prediction of postoperative overall survival (OS).

**Methods:**

A total of 115 CRC patients who underwent radical resection at the Affiliated Kunshan Hospital of Jiangsu University between 2018 and 2019 were retrospectively included. USP21 protein expression in tumor and adjacent normal tissues was assessed by immunohistochemistry. Prognostic factors were screened using univariate Cox regression followed by least absolute shrinkage and selection operator (LASSO) Cox regression to construct a multivariable nomogram. Model performance was evaluated using the concordance index (C-index), time-dependent ROC curves, calibration plots, and decision curve analysis (DCA). Patients were stratified into tertile-based risk groups for Kaplan–Meier survival validation.

**Results:**

USP21 expression was significantly higher in CRC tissues compared with adjacent normal tissues (*P* < 0.001) and was associated with depth of invasion, lymph node metastasis, TNM stage, and lymphovascular invasion (*P* < 0.05). Univariate Cox regression identified T, N, and M stages, LVI, PNI, and high USP21 expression as significant predictors of poor OS. LASSO-Cox regression retained five prognostic variables—USP21 expression, LVI, T stage, N stage, and M stage—for nomogram construction. Risk stratification based on nomogram-derived tertile cutoffs showed significant differences in OS among the low-, intermediate-, and high-risk groups (P < 0.001). The nomogram demonstrated strong predictive performance, with a C-index of 0.820 and time-dependent AUCs of 0.916 (3-year) and 0.854 (5-year). Calibration curves showed excellent agreement between predicted and observed survival, and DCA indicated substantial clinical net benefit.

**Conclusion:**

The USP21-based nomogram integrating key clinicopathological variables provides accurate individualized survival prediction and serves as a promising adjunctive tool for refining postoperative prognostic assessment and guiding clinical decision-making.

## 1 Introduction

Colorectal cancer (CRC) is the third most commonly diagnosed malignancy and the second leading cause of cancer-related deaths worldwide, with approximately 1.9 million new cases and over 900,000 deaths reported in 2022 (Bray et al., 2024). The TNM stage system developed by the American Joint Committee on Cancer (AJCC) remains the cornerstone for prognostic evaluation and treatment stratification in CRC (Weiser, 2018). However, substantial tumor heterogeneity leads to markedly different clinical outcomes and treatment responses among patients with the same TNM stage (Hari et al., 2013). Therefore, reliable biomarkers that complement traditional staging and refine individualized prognostication are urgently needed.

Ubiquitin-specific protease 21 (USP21), a deubiquitinating enzyme, has been shown to regulate the stability of multiple oncogenic proteins and plays tumor-promoting roles in breast cancer, gastric cancer, hepatocellular carcinoma, and lung cancer (Arceci et al., 2019; Li et al., 2018; Guo et al., 2021; Xu et al., 2020). Emerging evidence suggests that USP21 may also contribute to CRC progression (Nie et al., 2024; Lin and Lu, 2024; Yun et al., 2020; Shin et al., 2024). Nevertheless, the prognostic significance of USP21 protein expression in patient-derived CRC tissues has not been systematically investigated.

Nomograms integrate multiple prognostic variables to generate individualized risk predictions and have been widely applied in cancer prognostication, including gastric, colorectal, and breast cancers (Iasonos et al., 2008; Dong et al., 2020; Wang et al., 2022; Yang R. et al., 2023; Yu and Zhang, 2020). By combining clinicopathological and molecular features, nomograms often provide superior predictive accuracy and improved clinical applicability compared with traditional staging systems (Yang M. et al., 2023; Wang et al., 2024).

In this retrospective study, we evaluated the prognostic significance of USP21 protein expression in CRC specimens and incorporated USP21 into a multivariable prognostic nomogram for predicting 3- and 5-year overall survival (OS). Model performance was comprehensively assessed using the concordance index (C-index), time-dependent ROC, calibration curves, and decision curve analysis (DCA). We aimed to provide an individualized prognostic tool to support clinical strategy in CRC.

## 2 Materials and methods

### 2.1 Public data acquisition and bioinformatic analysis

The differential expression of the *USP21* gene between various malignant tumor tissues and their corresponding normal tissues was analyzed using the TIMER (Tumor Immune Estimation Resource) database (http://timer.cistrome.org/). Clinical information and corresponding RNA transcriptome sequencing data expression profiles for the colorectal cancer cohort were downloaded from The Cancer Genome Atlas (TCGA) database. The GSE83889 dataset, derived from the GPL10558 platform and containing RNA microarray expression profiles of 101 colorectal cancer samples and 35 matched normal samples, served as an independent dataset for external validation of differential expression.

### 2.2 Patient selection and data collection

A total of 115 CRC patients who underwent radical surgical resection at Affiliated Kunshan Hospital of Jiangsu University between June 2018 and December 2019 were retrospectively enrolled. Inclusion criteria were: (a) Clear pathological diagnosis of colorectal adenocarcinoma; (b) the patients had not been treated with radiotherapy, chemotherapy, molecular targeted therapy, or immunotherapy before the surgery; (c) patients without a history of other primary malignancies. Clinicopathological data included age, sex, tumor location, tumor size, differentiation grade, T stage, N stage, M stage, lymphovascular invasion (LVI), and perineural invasion (PNI), based on the AJCC 8th edition staging system. OS was defined as the interval from surgery to death or last contact. The study was approved by the institutional ethics committee.

### 2.3 Immunohistochemistry and scoring

USP21 protein expression was assessed by streptavidin–peroxidase immunohistochemistry using a rabbit monoclonal anti-USP21 antibody (1:400, Boster, A06639-1). Staining intensity and the percentage of positive tumor cells were evaluated independently by two pathologists in a double-blind manner. The final IHC score was calculated by combining intensity (0-3) and proportion scores (0-4), yielding a total score of 0–12. USP21 expression was categorized as low (≤4) or high (>4).

### 2.4 Variable selection and nomogram construction

Univariate Cox regression was performed to identify variables associated with OS. Significant variables were subjected to multivariate Cox regression. To improve model stability and reduce overfitting, least absolute shrinkage and selection operator (LASSO) Cox regression was further applied to variables significant in univariate analysis. Variables retained by LASSO were incorporated into a Cox regression model to construct the nomogram for predicting 3- and 5-year OS.

### 2.5 Model validation

Model performance was evaluated from four perspectives: (a) Discrimination: C-index with 1,000 bootstrap resamples. (b) Accuracy: AUCs for 3- and 5-year OS using time-dependent ROC curves. (c) Calibration: Bootstrap-corrected calibration curves comparing predicted and observed OS probabilities. (d) Clinical utility: Decision curve analysis (DCA) assessing net clinical benefit across threshold probabilities. Patients were stratified into low-, intermediate-, and high-risk groups using tertiles of nomogram-derived risk scores, and Kaplan–Meier curves were generated.

### 2.6 Statistical analysis

All analyses were conducted using R software (version 4.3.1). Categorical variables were compared using the *χ* ^*2*^ test or Fisher’s exact test. Continuous variables were analyzed using Student’s t-test or Mann–Whitney U test as appropriate. Survival differences were assessed using the log-rank test. Two-sided *P* < 0.05 was considered statistically significant.

## 3 Results

### 3.1 Public data analysis of USP21 expression and clinical significance

**FIGURE 1:**
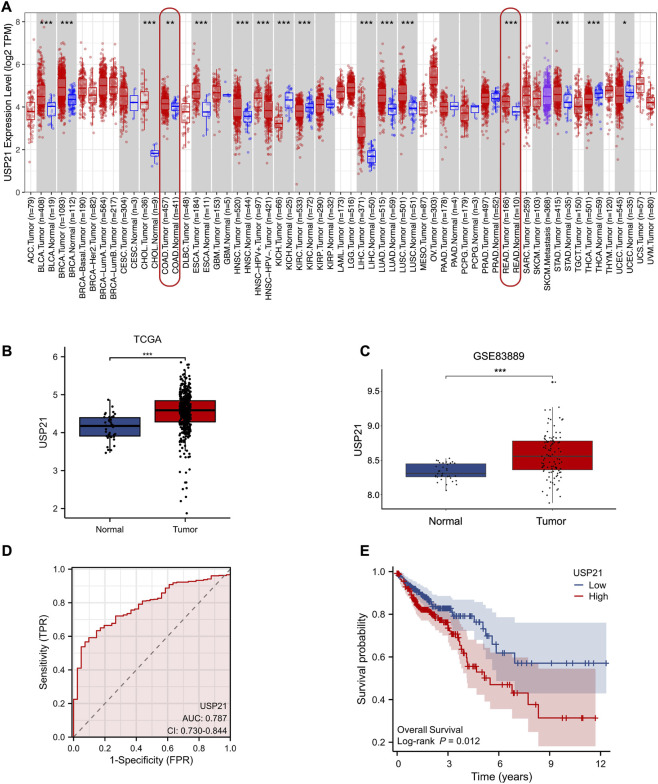
Expression and clinical significance of USP21 in CRC based on public database analysis. **(A)** USP21 transcriptional expression levels across various malignant tumor tissues based on TIMER database. **(B)** USP21 expression levels in CRC from TCGA database. **(C)** USP21 expression levels in the GSE83889 dataset. **(D)** ROC curve for USP21 expression in diagnosing TCGA-CRC. **(E)** Kaplan-Meier survival curves for overall survival (OS) stratified by USP21 expression in TCGA-CRC.

Pan-cancer analysis using the TIMER database revealed that the transcriptional expression level of USP21 was significantly upregulated in most malignant tumor tissues, including Breast Invasive Carcinoma (BRCA), Lung Adenocarcinoma (LUAD), and Stomach Adenocarcinoma (STAD). Notably, the upregulation was prominent in Colon Adenocarcinoma (COAD) and Rectal Adenocarcinoma (READ) (*P* < 0.01) (Figure 1A). Consistently, differential expression analysis based on the TCGA and the GSE83889 dataset showed that USP21 expression was significantly higher in colorectal tumor samples compared to normal tissues (*P* < 0.01) (Figures 1B,C). The Area Under the Curve (AUC) for USP21 in diagnosing CRC from the TCGA dataset was 0.787, suggesting its potential clinical diagnostic value as a biomarker (Figure 1D). Furthermore, overall survival (OS) prognostic analysis based on TCGA data demonstrated that high USP21 expression was significantly associated with unfavorable prognosis in CRC patients (*P* = 0.012) (Figure 1E).

### 3.2 USP21 expression and correlation with clinicopathological characteristics

To validate the findings from the public databases, the expression of USP21 protein was detected in 115 paired tumor tissues and adjacent normal tissues from CRC patients via immunohistochemistry (IHC). The baseline and clinicopathological characteristics of the 115 enrolled CRC patients are summarized in Table 1. The cohort consisted of 63 males (54.8%) and 52 females (45.2%), with 60 patients (52.2%) aged ≤65 years and 55 patients (47.8%) aged >65 years. Regarding disease progression, half of the patients presented with advanced tumors, including 54 patients (47.0%) in TNM stage III and 4 patients (3.5%) in stage IV. Correspondingly, a total of 70 patients (60.9%) received standard postoperative adjuvant therapy according to clinical guidelines. Following this baseline assessment, we evaluated the correlation between USP21 expression and these clinical features.

**TABLE 1:** Correlation between USP21 expression and clinicopathological characteristics.

Characteristic	Total (n = 115)	USP21 high (n = 71, %)	USP21 low (n = 44, %)	P-value
Gender				0.465
Male	63	37 (52.1)	26 (59.1)	
Female	52	34 (47.9)	18 (40.9)	
Age (years)				0.689
≤65	60	36 (50.7)	24 (54.5)	
>65	55	35 (49.3)	20 (45.5)	
Tumor location				**0.043**
Right	42	31 (43.7)	11 (25.0)	
Left/rectum	73	40 (56.3)	33 (75.0)	
Tumor size (cm)				0.088
<5	78	44 (62.0)	34 (77.3)	
≥5	37	27 (38.0)	10 (22.7)	
Differentiation				0.948
Moderate	71	44 (62.0)	27 (61.4)	
Poor	44	27 (38.0)	17 (38.6)	
Depth of invasion				**<0.001**
T1	4	0 (0.0)	4 (9.1)	
T2	17	4 (5.6)	13 (29.5)	
T3	75	49 (69.0)	26 (59.1)	
T4	19	18 (25.4)	1 (2.3)	
Lymph node metastasis				**0.006**
No	57	28 (39.4)	29 (63.6)	
Yes	58	43 (60.6)	15 (36.4)	
TNM stage				**<0.001**
I	16	3 (4.2)	13 (29.5)	
II	41	25 (35.3)	16 (36.4)	
III	54	39 (54.9)	15 (34.1)	
IV	4	4 (5.6)	0 (0.0)	
LVI				**<0.001**
No	76	38 (53.5)	38 (86.4)	
Yes	39	33 (46.5)	6 (13.6)	
PNI				0.077
No	104	61 (85.9)	43 (97.7)	
Yes	11	10 (14.1)	1 (2.3)	
Adjuvant therapy				**0.001**
No	45	19 (26.8)	26 (59.1)	
Yes	70	52 (73.2)	18 (40.9)	

Bold values indicate statistical significance (*P* < 0.05).

**FIGURE 2:**
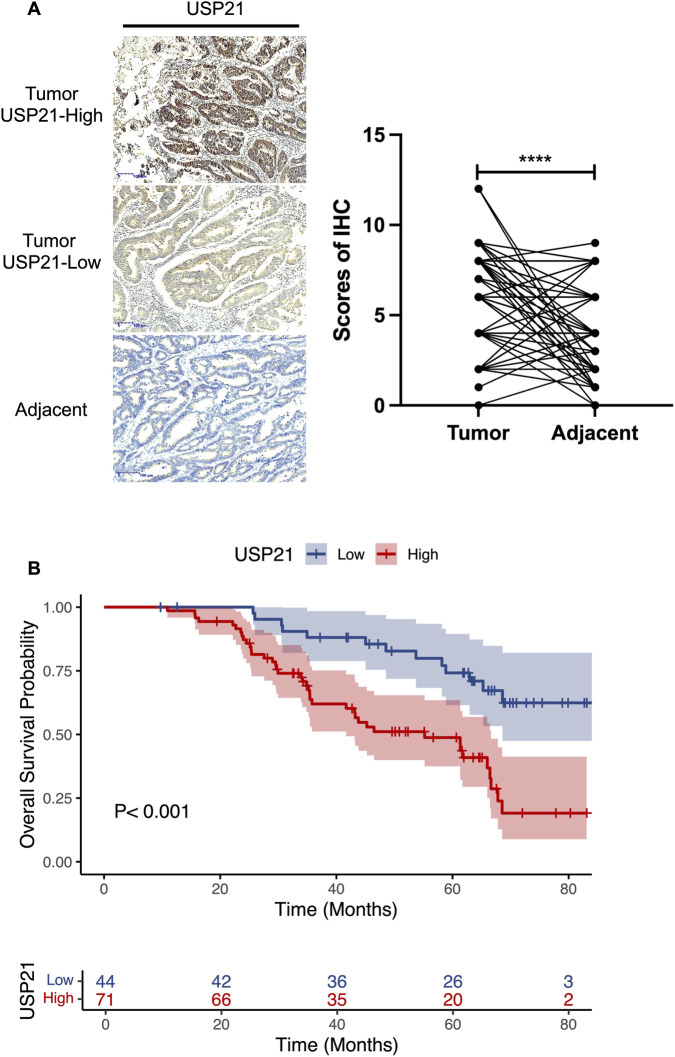
Upregulation of USP21 correlates with poor prognosis in CRC patients. **(A)** Representative immunohistochemical staining of USP21 in CRC tissues. **(B)** Kaplan–Meier survival curves for high and low USP21 expression groups.

The results demonstrated that the expression level of USP21 protein was significantly upregulated in CRC tumor tissues compared with their corresponding adjacent normal tissues (*P* < 0.001) (Figure 2A). Based on the predefined scoring system, the 115 CRC patients were divided into a high-expression group (n = 71) and a low-expression group (n = 44). Correlation analysis between USP21 expression and patient clinicopathological characteristics (Table 1) revealed that USP21 expression was significantly associated with tumor location (*P* = 0.043), depth of invasion (*P* < 0.001), lymph node metastasis status (*P* = 0.006), TNM stage (*P* < 0.001), and the presence of lymphovascular invasion (LVI) (*P* < 0.001). Conversely, USP21 expression showed no significant correlation with gender, age, tumor size, differentiation grade, or perineural invasion (PNI). Notably, the administration of adjuvant therapy was significantly more frequent in the USP21-high group compared to the USP21-low group (73.2% vs. 40.9%, *P* = 0.001), which is consistent with the advanced TNM stage observed in the USP21-high patients.

### 3.3 Univariate and multivariate cox regression analyses

Univariate Cox analysis identified T stage, N stage, M stage, LVI, PNI, and high USP21 expression as predictors of decreased OS (*P* < 0.05; Table 2). Kaplan-Meier analysis further showed significantly poorer OS in patients with high USP21 expression (*P* < 0.001; Figure 2B). In multivariate Cox analysis, only N1–2 stage and M1 stage remained independent prognostic factors (*P* < 0.001; Table 2).

**TABLE 2:** Univariate and multivariable cox regression analysis for overall survival.

Variable	Univariate analysis			Multivariable analysis		
	*HR*	95% CI	*P*- value	*HR*	95% CI	*P*- value
Gender						
Male	1.00					
Female	0.91	0.53–1.57	0.747			
Age (years)						
≤65	1.00					
>65	1.29	0.75–2.22	0.361			
Tumor location						
Right	1.00					
Left/rectum	0.77	0.44–1.35	0.364			
Tumor size (cm)						
<5	1.00					
≥5	1.45	0.82–2.55	0.201			
Differentiation						
Moderate	1.00					
Poor	1.65	0.96–2.85	0.071			
T stage						
T1-2	1.00			1.00		
T3	3.22	1.25–8.26	**0.015**	1.33	0.46–3.82	0.602
T4	15.98	5.27–48.42	**< 0.001**	2.32	0.57–9.39	0.239
N Stage						
N0	1.00			1.00		
N1	5.14	2.60–10.15	**< 0.001**	3.78	1.78–8.04	**< 0.001**
N2	8.92	4.28–18.61	**< 0.001**	4.75	2.01–11.20	**< 0.001**
M stage						
M0	1.00			1.00		
M1	34.27	8.96–131.12	**< 0.001**	11.79	2.81–49.41	**< 0.001**
LVI						
No	1.00			1.00		
Yes	3.88	2.20–6.83	**< 0.001**	1.64	0.84–3.21	0.151
PNI						
No	1.00			1.00		
Yes	2.72	1.27–5.82	**0.010**	0.81	0.35–1.90	0.630
USP21						
Low	1.00			1.00		
High	3.24	1.71–6.13	**< 0.001**	1.73	0.81–3.70	0.154

Bold values indicate statistical significance (*P* < 0.05).

### 3.4 LASSO-cox regression and nomogram development

**FIGURE 3:**
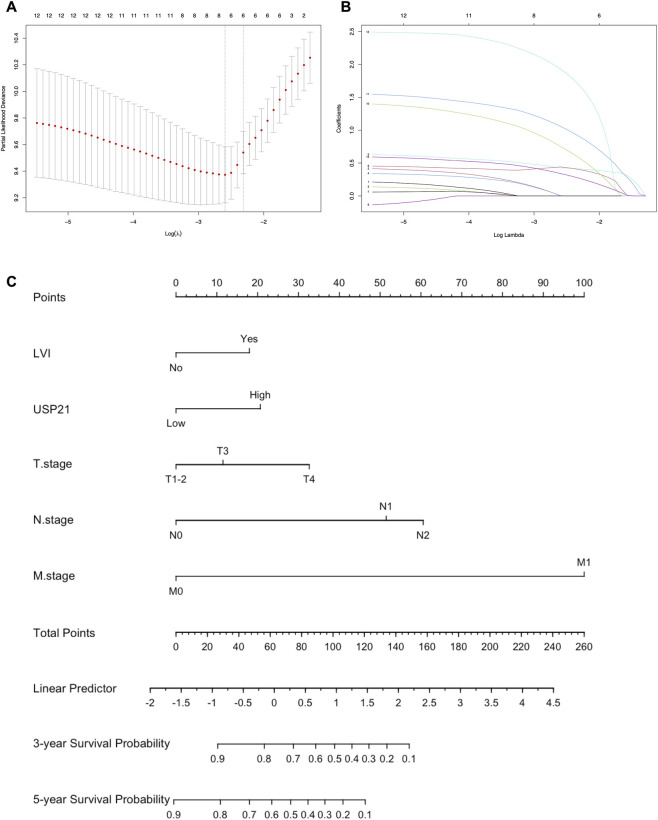
Variable selection by LASSO-Cox regression and nomogram construction for OS. **(A)** LASSO-Cox regression feature selection. **(B)** LASSO coefficient profiles. **(C)** Prognostic nomogram for predicting 3-year and 5-year overall survival in CRC patients.

To develop a concise and robust prognostic model, LASSO-Cox regression with 10-fold cross-validation was performed on variables that were significant in the univariate analysis. Using the one-standard-error (1-SE) criterion to select the optimal λ value (Figure 3A), five variables with non-zero coefficients were retained (Figure 3B). These prognostic factors—USP21 expression, LVI, T stage, N stage, and M stage—were subsequently incorporated into the construction of a nomogram to predict 3-year and 5-year OS in CRC patients (Figure 3C).

### 3.5 Risk stratification based on nomogram-derived risk scores

**FIGURE 4:**
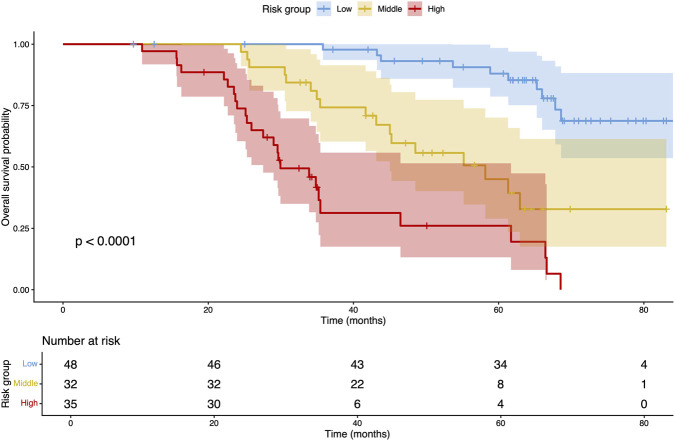
Kaplan–Meier survival curves for overall survival stratified by nomogram-derived risk groups.

Patients were stratified into low-, intermediate-, and high-risk groups according to tertiles of the nomogram-derived risk scores. Kaplan–Meier survival analysis demonstrated distinct and progressively worsening OS across the three groups (*P* < 0.001; Figure 4). Individuals in the high-risk group showed the poorest survival probability, whereas low-risk patients exhibited the most favorable outcomes.

### 3.6 Model performance evaluation

**FIGURE 5:**
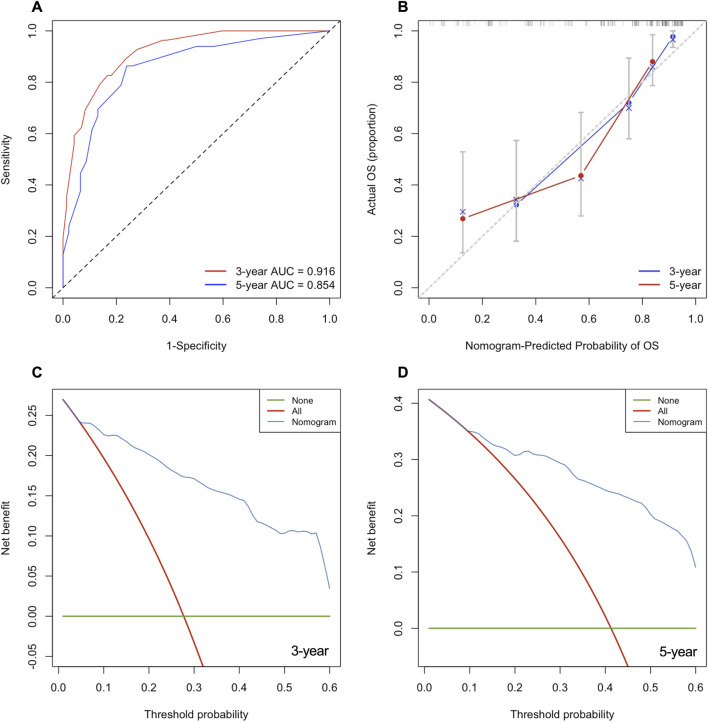
Performance and clinical utility evaluation of the prognostic nomogram. **(A)** Time-dependent receiver operating characteristic (ROC) curves for predicting 3- and 5-year OS. **(B)** Calibration curve for the nomogram. **(C,D)** Decision curve analysis (DCA) demonstrating the net clinical benefit of the nomogram at 3 and 5 years.

The nomogram demonstrated strong discriminative ability, achieving a C-index of 0.820 (95% CI: 0.773–0.867). Time-dependent ROC analysis showed excellent predictive performance, with a 3-year AUC of 0.916 and a 5-year AUC of 0.854 (Figure 5A). Calibration curves indicated a high level of agreement between predicted and observed OS probabilities at both 3 and 5 years (Figure 5B). Decision curve analysis (DCA) confirmed favorable net clinical benefit across a wide range of threshold probabilities at 3 and 5 years (Figures 5C,D).

## 4 Discussion

Currently, the application of standardized surgery and neoadjuvant/adjuvant strategies has significantly improved the clinical outcomes of most patients with colorectal cancer. For patients undergoing radical surgery for colorectal cancer, postoperative TNM stage remains an important factor in determining subsequent treatment modalities and evaluating prognosis (Delattre et al., 2022). Nevertheless, the current prognostic prediction methods still lack sufficient individualization and accuracy, and biomarkers with both sensitivity and specificity are still elusive.

A recent study developed a nomogram incorporating USP52 expression and TNM stage to predict colorectal cancer prognosis (Zhou et al., 2024). However, as it relied on public transcriptomic data rather than protein staining results, its translation into clinical application remains limited. In this study, we demonstrated that USP21 is markedly overexpressed in CRC tissues and is associated with multiple aggressive clinicopathological features, including deeper tumor invasion, lymph node metastasis, advanced TNM stage, and lymphovascular invasion. These findings reinforce a growing body of evidence suggesting that USP21 exerts oncogenic functions in solid tumors. Previous mechanistic investigations have shown that USP21 stabilizes oncogenic transcription factors and signaling molecules—such as ZEB1, Fra-1, and EGFR—thereby enhancing epithelial–mesenchymal transition, inflammatory signaling, and proliferative capacity (Lin and Lu, 2024; Yun et al., 2020; Shin et al., 2024). Our study extends these observations to patient-derived CRC tissues, providing clinical validation that elevated USP21 levels correlate with poorer overall survival.

Beyond establishing USP21 as a prognostic marker, this study systematically evaluated the relative contributions of clinicopathological and molecular features through a stepwise variable selection strategy. First, univariate Cox analysis was performed to comprehensively screen all potential predictors of survival. Next, multivariate Cox regression was applied to identify independent prognostic factors after adjusting for confounding variables; only N stage and M stage remained significant. However, multivariate Cox regression alone may overlook clinically meaningful variables due to collinearity, limited sample size, or nonlinear relationships. Because the oncogenic role of USP21 clinically manifests as advanced T/N stages and LVI, its prognostic variance is heavily absorbed by these dominant pathological factors in an unpenalized model. Therefore, to construct a stable and parsimonious predictive model, we further employed LASSO-Cox regression—a penalized regression method designed to reduce overfitting and improve model generalizability.

LASSO regression selected five variables with non-zero coefficients: USP21 expression, LVI, T stage, N stage, and M stage. Among them, LVI is a well-recognized high-risk factor for a poor prognosis in colorectal cancer (Zarbaliyev et al., 2024). LASSO is particularly advantageous in moderate-sized datasets such as ours because it handles multicollinearity effectively, prevents overfitting, and yields more reproducible models. Regression-based modeling strategies have also been widely applied in colorectal cancer research to identify independent clinical risk factors in different clinical settings (Zhu et al., 2024). The convergence of TNM parameters with USP21 and LVI underscores the biological and clinical relevance of these variables and supports their integration into a comprehensive prognostic framework. Additionally, the tertile-based risk stratification system clearly distinguished survival outcomes among low-, intermediate-, and high-risk groups, further enhancing the practical value of the model.

The resulting nomogram demonstrated excellent calibration, discrimination, and clinical usefulness. The C-index of 0.820 and high AUC values at 3 and 5 years reflect the model’s strong predictive accuracy. Calibration curves revealed close agreement between predicted and observed survival probabilities, indicating reliable risk estimation. Importantly, DCA confirmed meaningful net clinical benefit across a wide threshold range, suggesting that incorporating the nomogram into clinical practice may assist clinicians in postoperative counseling, surveillance planning, and treatment stratification.

Despite these strengths, several critical limitations must be heavily emphasized. First, the nomogram was constructed based on a relatively small, single-center cohort (n = 115) without a dedicated internal training-testing split or an independent external clinical validation cohort. Consequently, there is a high risk of model overfitting, and the generalizability of our predictive model remains uncertain. Although internal bootstrapping was utilized to calculate the C-index, it cannot substitute for true external validation. Since public databases like TCGA lack matched protein-level IHC data, future large-scale, multicenter prospective studies are strictly required to externally validate the robustness and clinical transportability of this integrated nomogram. Furthermore, molecular features known to influence CRC prognosis—such as MSI/MMR status and KRAS/NRAS/BRAF mutations—were not included due to unavailable data. Future studies integrating genomic markers with protein-level biomarkers such as USP21 may yield even more powerful prognostic tools. Prospective multicenter investigations are needed to validate the clinical utility of USP21 and to refine the predictive model for real-world application (Han et al., 2021).

In summary, this study identifies USP21 as a promising prognostic biomarker in CRC and highlights its association with aggressive characteristics. By integrating USP21 with key clinicopathological variables through a penalized regression framework, we developed a robust nomogram capable of providing individualized survival prediction. This model may support precision oncology approaches and guide postoperative therapeutic decisions in CRC patients.

## Data Availability

The raw data supporting the conclusions of this article will be made available by the authors, without undue reservation.
